# Nano-Enabled Fluorescence Switching: A Novel Strategy for PDGFRβ Detection and TKI Therapy Monitoring

**DOI:** 10.34133/research.1218

**Published:** 2026-03-24

**Authors:** Xin Fu, Jinyue Fan, Haoxiang Chen, Yuli Zheng, Yueqi Liu, Chao Zhang, Xiaolong Cao, Tingting Zuo

**Affiliations:** ^1^ College of Biological Sciences and Technology, Yili Normal University, Yining 835000, P.R. China.; ^2^ Department of Oncology, Zhujiang Hospital, Southern Medical University, Guangzhou 510282, P.R. China.; ^3^ Xinjiang Key Laboratory of Lavender Conservation and Utilization, Yining 835000, P.R. China.; ^4^ Translational Medicine Research Center, Zhujiang Hospital, Southern Medical University, Guangzhou 510282, P.R. China.; ^5^ Department of Anesthesiology, Zhujiang Hospital, Southern Medical University, Guangzhou, Guangdong 510282, P.R. China.

## Abstract

Determining platelet-derived growth factor receptor β (PDGFRβ) expression in biological specimens is pivotal for cancer diagnosis, drug development, and therapeutic monitoring. After tyrosine kinase inhibitor (TKI) therapy, altered PDGFRβ expression may correlate with treatment resistance mechanisms. Real-time, accurate detection of PDGFRβ levels pre- and post-TKI treatment holds substantial clinical value, as it enables therapeutic efficacy evaluation, resistance prediction, and timely regimen adjustment. However, the current repertoire of real-time technologies for precise PDGFRβ monitoring remains highly limited. Herein, we present a novel nanoprobe (Cy3-Gint4.T@BPNSs) for PDGFRβ detection based on a fluorescence quenching–recovery mechanism. Cy3-Gint4.T is a cyanine 3 (Cy3)-labeled aptamer with high specificity and strong selective binding affinity for PDGFRβ. Black phosphorus nanosheets (BPNSs) adsorb Cy3-Gint4.T via van der Waals forces to quench its fluorescence. Upon targeting PDGFRβ on cancer cells, the aptamer–receptor interaction outcompetes Cy3-Gint4.T’s binding to BPNSs, triggering its release and subsequent fluorescence restoration. Notably, the restored fluorescence intensity shows a direct correlation with cellular PDGFRβ expression, highlighting the nanoprobe’s potential for guiding tumor diagnosis and treatment. Critically, our data confirm that dynamic PDGFRβ expression changes induced by specific TKI therapies exhibit a proportional relationship with corresponding fluorescence intensity variations. This finding further supports an association between PDGFRβ expression dynamics and TKI resistance mechanisms, facilitating precise PDGFRβ monitoring and individualized therapeutic guidance.

## Introduction

Epidermal growth factor receptor (EGFR), a transmembrane receptor tyrosine kinase expressed in normal tissues, is frequently amplified or mutated in solid tumors such as epithelial cancer and glioblastoma (GBM), making it a key therapeutic target [1–7]. EGFR tyrosine kinase inhibitors (TKIs) block downstream signaling pathways by competitively binding the adenosine triphosphate (ATP)-binding site of EGFR, achieving important progress in GBM treatment [8,9]. However, acquired resistance to EGFR-TKIs remains a major obstacle in oncology [10–12]. While multiple genetic mechanisms of TKI resistance have been identified in various cancers, nongenetic resistance mechanisms, such as derepression of platelet-derived growth factor receptor β (PDGFRβ) transcription, have also been shown to drive acquired resistance to EGFR-TKIs in GBM patients [13–18]. PDGFRβ, a member of the tyrosine kinase receptor family, functions as a transmembrane glycoprotein localized on the cell surface [19–21]. It is activated upon binding to various growth factors [22,23]. Activation and autophosphorylation of PDGFRβ initiate a series of intracellular signaling cascades that regulate key cellular functions, including proliferation, migration, survival, and angiogenesis [24–27]. Abnormal activation, mutations, or overexpression of the PDGFRβ gene have been identified in various cancers, such as GBM, gastrointestinal stromal tumors, and lung cancer [28–30]. Abnormal PDGFRβ signaling promotes tumor growth, invasion, metastasis, and angiogenesis, making it a therapeutic target for many cancers [31–33]. Therefore, real-time, accurate monitoring of PDGFRβ expression levels before and after EGFR-TKIs treatment holds important clinical guidance value for evaluating treatment efficacy, predicting resistance, and optimizing therapeutic strategies, such as the timely incorporation of PDGFRβ inhibitors.

Recent studies have further established the PDGFRβ signaling pathway as a central player in mediating acquired TKI resistance. Upon inhibition of primary targets like EGFR by TKIs, tumor cells frequently activate alternative receptor tyrosine kinases through a mechanism known as “bypass signaling”, with PDGFRβ being a prominent member [34,35]. The up-regulation of PDGFRβ not only reactivates key downstream prosurvival cascades such as phosphatidylinositol 3-kinase (PI3K)/AKT and mitogen-activated protein kinase (MAPK)/extracellular signal–regulated kinase (ERK) but also promotes epithelial–mesenchymal transition (EMT) and the maintenance of cancer stem cell properties, thereby directly driving the resistant phenotype [36–38]. Furthermore, clinical evidence indicates elevated PDGFRβ expression in tumor tissues from patients with non-small cell lung cancer and GBM following treatment with gefitinib or erlotinib, correlating with shorter progression-free survival [18,39]. Consequently, the dynamic expression of PDGFRβ serves as a sensitive indicator of tumor adaptive response under TKI pressure, and its trajectory is closely linked to therapeutic outcomes, making it a clinically valuable biomarker for real-time monitoring.

Conventional PDGFRβ detection methods, including Western blotting (WB), enzyme-linked immunosorbent assay (ELISA), and immunohistochemistry (IHC), are time-consuming, involve cumbersome procedures, and require chromogens, fluorophores, or enzyme labels [40–43]. Furthermore, WB and IHC require highly skilled personnel for precise execution. Conventional techniques such as ELISA and WB, while highly sensitive and specific, are inherently endpoint assays that require multistep processing, prolonged incubation times (typically hours to days), and specialized equipment operated by trained personnel [44,45]. These limitations hinder real-time, dynamic, and convenient monitoring of PDGFRβ expression, particularly in clinical practice where frequent assessment of TKI treatment efficacy and resistance mechanisms is required. Thus, we urgently need to develop a simple, direct, efficient, and dynamically responsive PDGFRβ detection method.

**Fig. 1.**
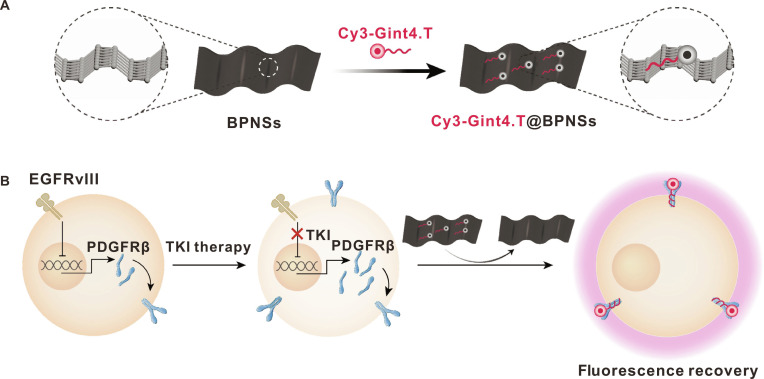
Schematic illustration of the Cy3-Gint4.T@BPNSs principle. (A) Schematic representation of Cy3-Gint4.T loading onto BPNSs. (B) Functional schematic of Cy3-Gint4.T@BPNSs, where the degree of fluorescence recovery reflects the expression level of PDGFRβ: In the presence of PDGFRβ, Cy3-Gint4.T specifically binds to PDGFRβ and dissociates from BPNSs, leading to the recovery of Cy3 fluorescence.

Black phosphorus nanosheets (BPNSs) are a type of fluorescent quenching nanomaterial that is widely used due to its graphene-like layered structure, high specific surface area, and abundance of active sites [46–50]. They also exhibit high carrier mobility, excellent optical properties, good biocompatibility, and ease of functionalization [51–56]. Compared to traditional graphene oxide, BPNSs offer several distinct advantages, including a tunable direct bandgap, stronger fluorescence quenching efficiency via efficient Förster resonance energy transfer (FRET) and photoinduced electron transfer (PET) mechanisms, and superior biodegradability, which reduces long-term toxicity concerns. Furthermore, the puckered lattice structure of BPNSs provides abundant active sites for biomolecule adsorption via van der Waals interactions, enabling high-density aptamer loading and enhanced sensing sensitivity. Notably, BPNSs also exhibit near-infrared (NIR) optical responsiveness and photothermal properties, expanding their potential for theranostic applications beyond fluorescence sensing [57–64]. In this study, we propose an innovative strategy for visualizing and detecting PDGFRβ expression using a multilayered BPNS-based nanoscale quenching–recovery detector (Cy3-Gint4.T@BPNSs) loaded with cyanine 3 (Cy3)-labeled PDGFRβ aptamer (Cy3-Gint4.T). Aptamers are adsorbed onto the surface of the BPNSs via van der Waals forces acting between the nucleobases and the phosphorene structure, resulting in efficient fluorescence quenching. As shown in Fig. 1A, Cy3-labeled PDGFRβ-targeting aptamers (Cy3-Gint4.T) undergo effective fluorescence quenching upon binding to BPNSs. Subsequent recognition of PDGFRβ on cancer cell surfaces triggers the release of Cy3-Gint4.T from BPNSs as a consequence of the high-affinity interaction between the aptamer and PDGFRβ, leading to fluorescence signal recovery (Fig. 1B). This fluorescence recovery is a reliable indicator of the presence of PDGFRβ on the surfaces of cancer cells, demonstrating the potential of the system for targeted cancer diagnosis and therapeutic applications. We anticipate that Cy3-Gint4.T@BPNSs will enable intuitive, dynamic monitoring of PDGFRβ expression levels within tumors, providing a powerful imaging tool for the guidance of therapeutic strategies and the improvement of patient prognosis.

## Results and Discussion

### Synthesis and characterization of Cy3-Gint4.T@BPNSs

**Fig. 2.**
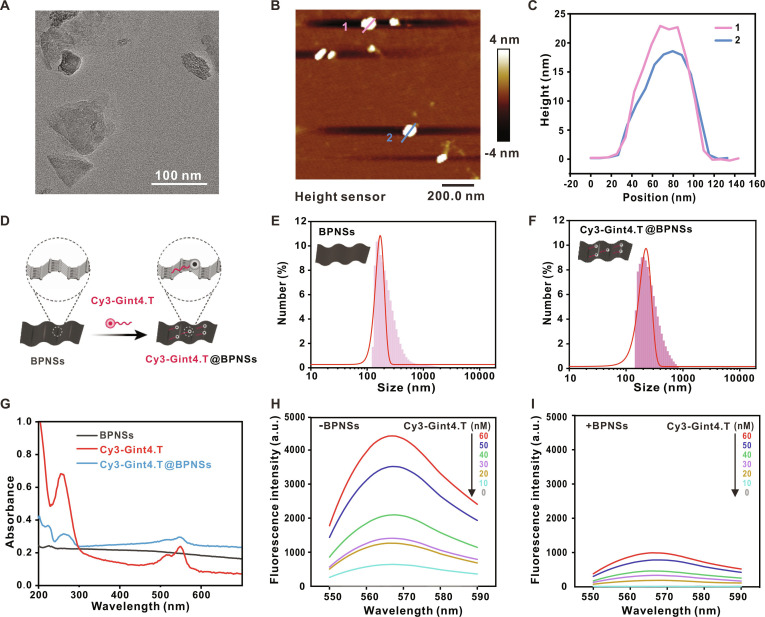
Characterization of Cy3-Gint4.T@BPNSs. (A) TEM image of BPNSs. (B) AFM image of BPNSs. (C) Height distribution profiles corresponding to the line segments in (B). (D) Schematic illustration of Cy3-Gint4.T loading onto BPNSs for the preparation of Cy3-Gint4.T@BPNSs. (E and F) Distribution curves of hydrodynamic diameters corresponding to BPNSs and Cy3-Gint4.T@BPNSs, respectively. (G) UV–Vis absorption profiles of Cy3-Gint4.T, BPNSs, and Cy3-Gint4.T@BPNSs. (H and I) Fluorescence spectra of Cy3-Gint4.T, evaluated via concentration gradients in BPNS-free and BPNS-containing systems, respectively.

BPNSs are a distinct and promising subclass of 2-dimensional materials consisting of one or a few layers of phosphorus atoms. Transmission electron microscopy (TEM) and atomic force microscopy (AFM) were used to characterize the dimensions and morphology of synthesized BPNSs. As demonstrated in Fig. 2A and B, the nanosheets exhibited superimposed wrinkles with disparate thicknesses. TEM imaging indicated an average lateral size of approximately 125 nm (Fig. 2A). AFM height profile analysis (Fig. 2C) recorded thicknesses of 22.9 and 18.6 nm for 2 representative lines (line 1 and line 2, respectively). These collective results confirm the successful preparation of high-quality, ultrathin BPNSs suitable for subsequent functionalization and bioimaging applications. Cy3-Gint4.T was purchased from Sangon Biotech. The Cy3 fluorescent molecules and Gint4.T were linked through covalent reactions, no significant shift was observed in its fluorescence emission spectrum, and the maximum emission wavelength remained almost unchanged (Fig. S1). This result suggests that aptamer modification exerts only a minor influence on the photophysical properties of the Cy3 dye. The fluorescence intensity of Cy3-Gint4.T was tested under conditions of different pH values. The results showed that there was no significant difference under different pH conditions, indicating that it is an effective fluorescent indicator molecule (Fig. S2).

The optimal loading ratio between the fluorescent aptamer Cy3-Gint4.T and BPNSs was determined via a gel retardation assay. Quantitative analysis of gel images (Figs. S3 and S4) revealed that at a constant BPNS concentration of 0.2 mg/ml, free RNA signals disappeared when the Cy3-Gint4.T concentration was below 40 nM, indicating near-complete adsorption of the aptamer onto the nanosheets. Successful complex formation was further evidenced by efficient fluorescence quenching of Cy3-Gint4.T upon adsorption onto the BPNS surface (Fig. 2D). The quenching mechanism is primarily attributed to FRET and PET from the fluorophore to the conductive BPNSs, facilitated by close proximity and strong electronic coupling between the aptamer nucleobases and the phosphorene lattice. Upon target recognition, the higher affinity interaction between Cy3-Gint4.T and PDGFRβ competitively displaces the aptamer from BPNSs, leading to fluorescence recovery. This displacement is energetically favored due to the specific conformational change and binding energy of the aptamer–receptor complex, which exceeds the adsorption energy between the aptamer and BPNSs [65–67]. Dynamic light scattering (DLS) analysis provided additional validation: The average hydrodynamic diameter of normal BPNSs was about 160 nm (Fig. 2E), 44 nm smaller than that of Cy3-Gint4.T@BPNSs (Fig. 2F), consistent with surface conjugation. Ultraviolet–visible (UV–Vis) absorption spectroscopy corroborated the presence of Cy3-Gint4.T in the nanocomposite, as the characteristic 260-nm absorption peak of the aptamer was retained in Cy3-Gint4.T@BPNSs (Fig. 2G). To quantify the quenching efficiency, the fluorescence emission of free Cy3-Gint4.T was first examined, showing a concentration-dependent increase in intensity with a characteristic emission peak between 565 and 575 nm (Fig. 2H). Upon the addition of BPNSs, a marked reduction in fluorescence intensity was detected (Fig. 2I). The most significant quenching effect emerged when the concentration of Cy3-Gint4.T was below 40 nM, aligning with the gel retardation data and verifying efficient loading within this concentration range.

The stability of the Cy3-Gint4.T@BPNSs complex was assessed by analyzing filtrates collected over time at room temperature. No characteristic Cy3-Gint4.T absorption peak was detected in any sample (Fig. S5), indicating no significant dissociation of the aptamer from the nanosheets. In addition, we incubated Cy3-Gint4.T@BPNSs at different temperatures (25 and 37 °C) for the same period and measured the fluorescence intensity. No significant difference was observed, further demonstrating the stability of Cy3-Gint4.T@BPNSs (Fig. S6). Furthermore, a CCK-8 assay demonstrated good biocompatibility of the nanocomplex (Fig. S7). In summary, these results demonstrate the successful production of Cy3-Gint4.T@BPNSs, highlighting their excellent fluorescence quenching capability, stability, and biosafety.

### Responsiveness of Cy3-Gint4.T@BPNSs to PDGFRβ after TKI treatment

**Fig. 3.**
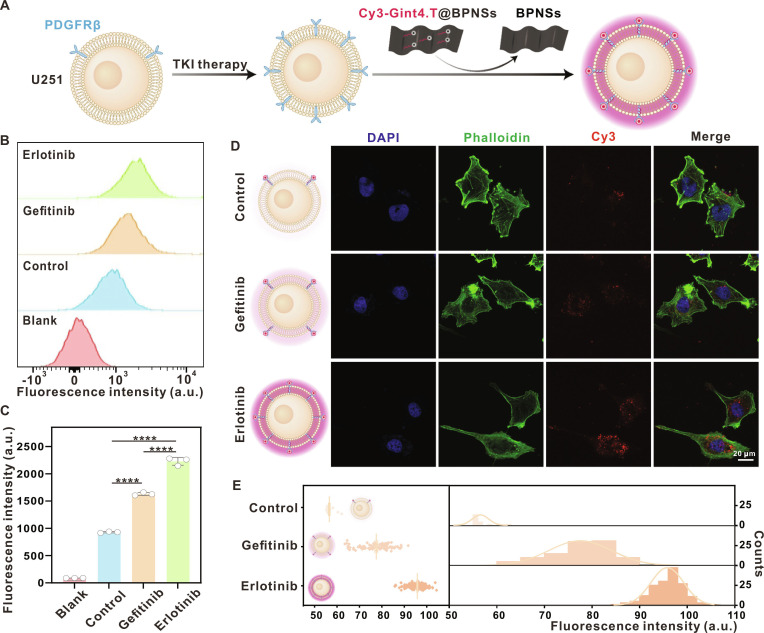
Cy3-Gint4.T@BPNSs responds to PDGFRβ expression in U251 cells. (A) Schematic of PDGFRβ detection via Cy3-Gint4.T@BPNSs. (B) Flow cytometry profiles of PDGFRβ antibody fluorescence (Blank: unstained; Control: untreated; Gefitinib/Erlotinib: TKI-treated). (C) Quantitative analysis of PDGFRβ antibody fluorescence (****P < 0.0001). (D) CLSM images of TKI-pretreated U251 cells incubated with Cy3-Gint4.T@BPNSs (scale bar, 20 μm). (E) Quantitative Cy3 fluorescence intensity from (D).

As previously reported, PDGFRβ amplification occurs in approximately half of primary glioma cases; here, we selected the U251 cells to evaluate the responsiveness of Cy3-Gint4.T@BPNSs to PDGFRβ expression (Fig. 3A). Flow cytometry analysis confirmed basal PDGFRβ expression on U251 cells, as well as its modulation following treatment with EGFR-TKIs (gefitinib and erlotinib) (Fig. 3B). Quantitative results revealed a significant up-regulation of PDGFRβ expression after TKI treatment, with a more pronounced increase in erlotinib-treated group compared with gefitinib-treated group (Fig. 3C). Correspondingly, when TKI-pretreated U251 cells were incubated with Cy3-Gint4.T@BPNSs (under light-free conditions), confocal laser scanning microscopy (CLSM) showed an apparent recovery of Cy3 fluorescence (Fig. 3D). Quantification of the Cy3 signal further demonstrated enhanced fluorescence intensity after TKI treatment (Fig. 3E), in agreement with the elevated PDGFRβ expression.

**Fig. 4.**
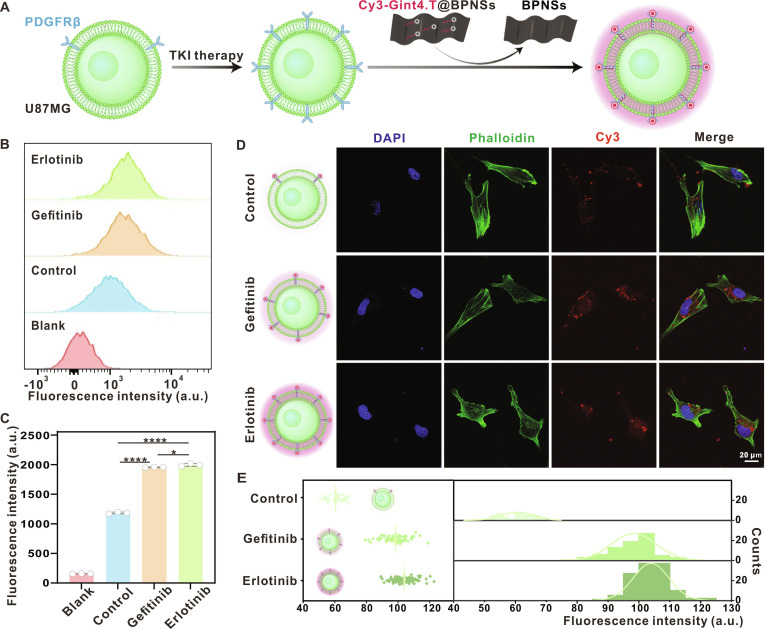
Cy3-Gint4.T@BPNSs responds to PDGFRβ expression in U87MG cells. (A) Schematic illustration depicting PDGFRβ detection in U87MG cells using Cy3-Gint4.T@BPNSs. (B) Flow cytometry profiles showing the fluorescence intensity of PDGFRβ-targeting antibodies in U87MG cells. (C) Quantitative analysis of PDGFRβ antibody fluorescence (*P < 0.05, ****P < 0.0001). (D) CLSM images of TKI-pretreated U87MG cells after incubation with Cy3-Gint4.T@BPNSs (scale bar, 20 μm). (E) Quantitative analysis of Cy3 fluorescence intensity corresponding to (D).

To further validate the target responsiveness and generalizability of Cy3-Gint4.T@BPNSs, we performed parallel experiments using U87MG cells (Fig. 4A). Flow cytometry confirmed basal PDGFRβ expression in U87MG cells, and its expression was significantly up-regulated following treatment with TKIs (Fig. 4B). Notably, quantitative analysis (Fig. 4C) revealed that both erlotinib and gefitinib induced a substantial up-regulation of PDGFRβ levels (65% and 69%, respectively), compared to the control. Consistently, fluorescence recovery assays under identical incubation conditions showed enhanced Cy3 signals in TKI-pretreated U87MG cells, with CLSM images (Fig. 4D) and quantitative analysis (Fig. 4E) confirming strengthened fluorescence recovery in TKI-treated groups. Collectively, these results demonstrate the up-regulation of PDGFRβ in GBM upon EGFR-TKI treatment, establishing Cy3-Gint4.T@BPNSs as a robust, responsive probe for PDGFRβ detection.

### Time-dependent responsiveness of Cy3-Gint4.T@BPNSs to PDGFRβ after TKI treatment

**Fig. 5.**
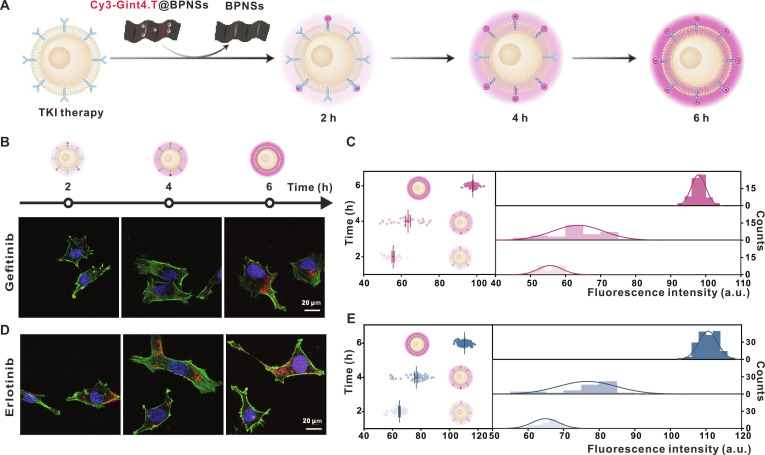
Time-dependent responsiveness of Cy3-Gint4.T@BPNSs in TKI-pretreated U251 cells. (A) Schematic illustration of the time-dependent interaction between Cy3-Gint4.T@BPNSs and TKI-pretreated U251 cells (incubation durations: 2, 4, and 6 h). (B and C) Representative CLSM images and quantitative fluorescence intensity analysis of gefitinib-pretreated U251 cells after 2, 4, and 6 h (scale bar, 20 μm). (D and E) Representative CLSM images and corresponding quantitative evaluations of fluorescence intensity in U251 cells subjected to erlotinib pretreatment for 2, 4, and 6 h, respectively (scale bar, 20 μm).

We next assessed the time-dependent responsiveness of Cy3-Gint4.T@BPNSs. TKI-pretreated U251 cells were incubated with Cy3-Gint4.T@BPNSs for 2, 4, or 6 h under light-free conditions (Fig. 5A). CLSM images and fluorescence intensity analysis (Fig. 5B and D) showed a progressive enhancement of Cy3 fluorescence signal over extended incubation periods, consistent with the time-dependent binding of Cy3-Gint4.T to up-regulated PDGFRβ.

**Fig. 6.**
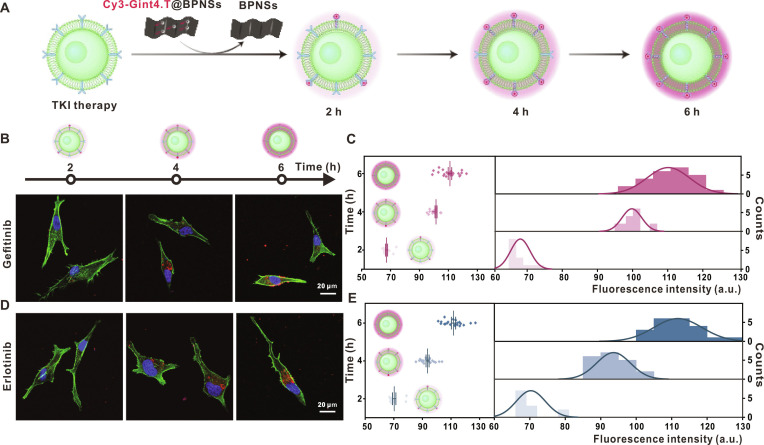
Time-dependent responsiveness of Cy3-Gint4.T@BPNSs in TKI-pretreated U87MG cells. (A) Schematic illustration of the time-dependent binding process between Cy3-Gint4.T@BPNSs and TKI-pretreated U87MG cells at different incubation durations. (B and C) Representative CLSM images and quantitative fluorescence intensity analysis of gefitinib-pretreated U87MG cells after 2, 4, and 6 h (scale bar, 20 μm). (D and E) Representative CLSM images and quantitative fluorescence intensity analysis of erlotinib-pretreated U87MG cells after 2, 4, and 6 h (scale bar, 20 μm).

This time-course experiment was subsequently replicated in U87MG cells (Fig. 6A). Consistent with the results in U251 cells, confocal fluorescence imaging (Fig. 6B and D) and quantitative analysis (Fig. 6C and E) demonstrated a progressive enhancement of fluorescence signal as incubation time extended, confirming the time-dependent responsiveness of Cy3-Gint4.T@BPNSs across different glioma cell lines.

### Cy3-Gint4.T@BPNSs as a specific nanodevice for PDGFRβ receptor monitoring

**Fig. 7.**
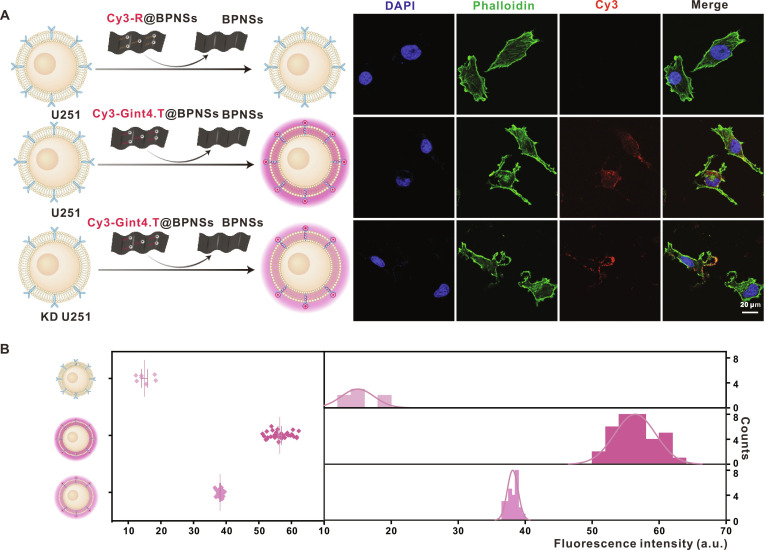
Validation of the target specificity of Cy3-Gint4.T@BPNSs for PDGFRβ detection in U251 cells. (A) Schematic illustration and CLSM images of Cy3-R@BPNSs, Cy3-Gint4.T@BPNSs, and Cy3-Gint4.T@BPNSs with KD U251 cells (scale bar, 20 μm). (B) Quantitative assessment of Cy3 fluorescence intensity corresponding to the CLSM images in (A).

To confirm the target specificity of Gint4.T, a random single-stranded RNA sequence (Cy3-R) of equal nucleotide length was employed instead of Gint4.T to develop a control nanoprobe (Cy3-R@BPNSs). As expected, incubation of Cy3-R@BPNSs with U251 cells showed no significant fluorescence recovery, whereas considerable fluorescence recovery was observed following incubation with Cy3-Gint4.T@BPNSs under identical conditions, thereby indicating the indispensable role of Gint4.T in the specific detection of PDGFRβ. To corroborate this specificity further, the expression of the PDGFRβ receptor was knocked down in U251 cells (KD U251), which were then incubated with Cy3-Gint4.T@BPNSs (Fig. 7A and B). A marked decrease in fluorescence recovery was detected in KD U251 cells compared to U251 cells, providing additional evidence of targeted PDGFRβ receptor recognition by Cy3-Gint4.T@BPNSs. Taken together, these experimental observations and statistical evaluations conclusively demonstrate the specific binding of Cy3-Gint4.T@BPNSs to PDGFRβ while minimizing contributions from nonspecific interactions.

### Accurate monitoring capacity of Cy3-Gint4.T@BPNSs for PDGFRβ dynamics during TKI therapy

**Fig. 8.**
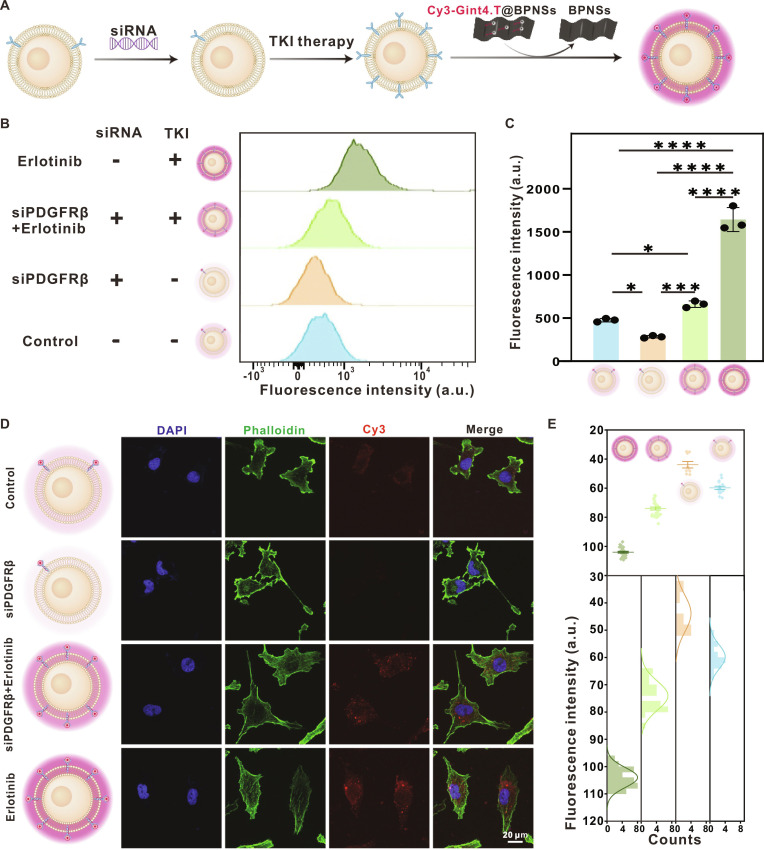
The ability of Cy3-Gint4.T@BPNSs to correctly monitor PDGFRβ dynamics in the U251 cell line during TKI therapy. (A) Schematic of PDGFRβ detection following PDGFRβ siRNA-mediated knockdown and/or TKI (erlotinib) treatment in U251 cells. (B) Flow cytometry histograms of PDGFRβ-associated fluorescence intensity in U251 cells from the blank, control, siPDGFRβ, siPDGFRβ + erlotinib, and erlotinib-treated groups. (C) Quantitative assessment of fluorescence intensity from (B) (*P < 0.05, ***P < 0.001, and ****P < 0.0001). (D) CLSM images of Cy3-Gint4.T@BPNSs-treated U251 cells across all experimental groups (scale bar, 20 μm). (E) Statistical analysis of Cy3 fluorescence intensity corresponding to (D).

To assess the capacity of Cy3-Gint4.T@BPNSs to monitor PDGFRβ dynamics during TKI therapy, PDGFRβ expression in U251 cells was suppressed via small interfering RNA (siRNA)-mediated knockdown using 3 distinct siRNA molecules (siRNA-1, siRNA-2, and siRNA-3). Flow cytometry with specific fluorescent antibodies (Fig. S8) and quantitative analysis (Fig. S9) evaluated knockdown efficiency, leading to the selection of siRNA-3 for subsequent experiments. We established 4 experimental groups: untreated control, siPDGFRβ alone, erlotinib (TKI) alone, and siPDGFRβ + erlotinib combination (Fig. 8A). Flow cytometry (using fluorescently labeled antibodies) and quantitative analyses demonstrated that PDGFRβ levels on the surface of siPDGFRβ-treated U251 cells were significantly reduced by ~40% relative to control cells. Notably, the siPDGFRβ + erlotinib group exhibited ~2-fold higher fluorescence than the siPDGFRβ-only group, suggesting partial restoration/compensation of PDGFRβ expression or accessibility upon TKI exposure. In contrast, these results indicate that TKI treatment rapidly up-regulates PDGFRβ receptor activity (Fig. 8B and C). Confocal microscopy imaging further confirmed that the fluorescence changes in Cy3-Gint4.T@BPNSs-treated cells (Fig. 8D and E) were consistent with flow cytometry results: The erlotinib alone group exhibited robust Cy3 signals (reflecting elevated PDGFRβ levels), whereas the siPDGFRβ + erlotinib combination group showed attenuated fluorescence (due to PDGFRβ knockdown). Collectively, these data validate that Cy3-Gint4.T@BPNSs enables dynamic monitoring of PDGFRβ and visual tracking of tumor resistance-related alterations during TKI therapy.

### PDGFRβ-mediated TKI resistance reversal and therapeutic efficacy in U251 cells

**Fig. 9.**
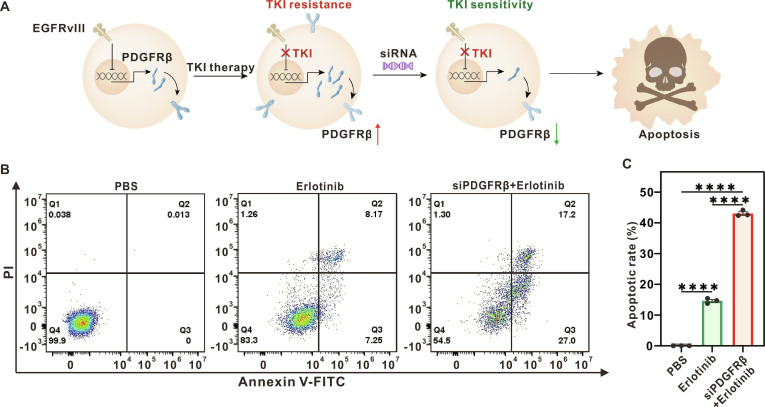
PDGFRβ silencing restores sensitivity to EGFR-TKI in TKI-resistant cells. (A) Schematic illustration of TKI resistance regulation via PDGFRβ. (B) Representative flow cytometry plots demonstrating apoptosis induction in U251 cells following exposure to PBS (control group), erlotinib alone, or a combined regimen of erlotinib and siPDGFRβ. (C) Quantitative analysis of apoptotic rates across groups (****P < 0.0001).

U251 cells (expressing EGFRvIII) exhibit TKI resistance due to PDGFRβ up-regulation following TKI therapy. To investigate whether suppressing PDGFRβ could restore TKI sensitivity, we performed siRNA-mediated PDGFRβ knockdown, which was hypothesized to lead to increased cell death (Fig. 9A). We evaluated the capacity of erlotinib alone versus PDGFRβ knockdown combined with erlotinib to induce apoptosis in U251 cells via Annexin V-fluorescein isothiocyanate (FITC)/propidium iodide (PI) staining. As demonstrated in Fig. 9B and C, the combination of PDGFRβ knockdown and erlotinib significantly enhanced the toxicity of the treatment against U251 cells in comparison to erlotinib monotherapy. The combination treatment resulted in an apoptotic rate of 44% in the cells, which was more than 2 times the rate observed in cells treated with erlotinib alone. We performed a comparative analysis of the half-maximal inhibitory concentration (IC_50_) values for U251 cells treated with erlotinib alone versus PDGFRβ knockdown combined with erlotinib. Analysis of the IC_50_ results revealed a distinct reduction in IC_50_ values following PDGFRβ knockdown, indicating greater drug potency and corresponding to attenuated TKI resistance in U251 cells (Fig. S10). These findings collectively demonstrate that PDGFRβ silencing effectively reverses TKI resistance and enhances the therapeutic efficacy of erlotinib in U251 cells.

## Conclusion

Cy3-Gint4.T@BPNSs, a nanoprobe for PDGFRβ detection based on the quenching–recovery mechanism, was successfully fabricated by loading Cy3-labeled PDGFRβ aptamer (Cy3-Gint4.T) onto BPNSs. Due to the high affinity between Gint4.T and PDGFRβ, Cy3-Gint4.T@BPNSs specifically recognizes PDGFRβ on cancer cells, enabling fluorescence-labeled visualization that positively correlates with TKI resistance. Our findings demonstrate that Cy3-Gint4.T@BPNSs fluorescence intensity directly reflects PDGFRβ expression levels, providing an intuitive readout of PDGFRβ status. Importantly, we validated the probe’s specificity for PDGFRβ and its ability to dynamically track PDGFRβ changes during therapeutic intervention. This Cy3-Gint4.T@BPNSs-based recognition and visual detection strategy offers a promising approach for early tumor diagnosis and therapeutic monitoring.

The Cy3-Gint4.T@BPNSs nanoprobe developed in this study demonstrates significant clinical translational potential for tumor diagnosis and treatment guidance. This probe enables real-time, dynamic monitoring of PDGFRβ expression changes, providing an intuitive fluorescence imaging tool for assessing therapeutic efficacy and early detection of resistance during TKI therapy. Moreover, the nanoprobe exhibits good biocompatibility and stability, making it suitable for further development as an in vivo imaging or liquid biopsy tool to advance personalized precision medicine and assist clinicians in identifying resistance trends at an early stage and enabling timely adjustment of treatment regimens. Future studies will focus on validating the system in animal models and clinical samples to accelerate its translational application. Clinically, this would allow real-time adjustments to medication regimens based on PDGFRβ expression status, thereby optimizing therapeutic outcomes.

## Methods

### Synthesis and characterization of Cy3-Gint4.T@BPNSs

Table S1 provides the sequence information for Cy3-Gint4.T and Cy3-R. BPNSs were obtained from Nanjing MKNANO Tech. Co. Ltd. To preclude re-agglomeration, the BPNSs were subjected to sonication in ice water for 5 min via a probe sonicator. The sonicator operated at a frequency of 20 Hz and an output power of 300 W, with a programmed cycle of 5 s on alternating with 2 s off. Cy3 is a carbocyanine dye widely used in fluorescence labeling due to its strong emission in the orange-red region (565 to 575 nm). Cy3-Gint4.T, a Cy3-labeled aptamer that exhibits high specificity for PDGFRβ, was employed to enable fluorescence-based detection and imaging. Cy3-Gint4.T@BPNSs were formed by mixing 40 nM Cy3-Gint4.T with 0.2 mg/ml of BPNSs in the dark for 5 min. The BPNS size was determined by AFM (Multimode 8, Bruker), and the structure was characterized by TEM (JEM-2100, Japan). The height distribution of BPNSs was quantified using NanoScope Analysis 2.0 software, which analyzed the AFM images statistically. Nanoparticle size was determined via DLS (Litesizer), and UV–Vis absorption spectra were acquired using a UV–Vis spectrophotometer (UV5Nano model).

### Stability of Cy3-Gint4.T in PBS at different pH

Cy3-Gint4.T was diluted to 40 nM (consistent with the loading concentration in the nanoprobe) in phosphate-buffered saline (PBS) at pH 7.4 and pH 6.0 (adjusted with 1 M HCl). The solutions were incubated at 37 °C, and 100-μl aliquots were taken at 2, 4, 6, 8, 10, and 12 h. The fluorescence intensity of each sample was quantified using a microplate reader (Bio-Tek, SYNERGY H1, USA) with an excitation wavelength of 520 nm and emission wavelength range of 565 to 575 nm. All experiments were performed in 3 independent biological replicates, and data were expressed as mean ± standard deviation (SD).

### CCK-8 assay

Normal human astrocyte (NHA) cells (3 × 10^4^) were seeded in 96-well plates (*n* = 3). Following cell adhesion, the culture medium was discarded, followed by the addition of Dulbecco’s modified Eagle’s medium (DMEM)–Cy3-Gint4.T@BPNSs mixtures at varied proportions to each well, which ensured the attainment of target Cy3-Gint4.T@BPNSs concentrations ranging from 0 to 0.2 mg/ml (0, 0.0125, 0.025, 0.05, 0.1, and 0.2 mg/ml). The amount of Cy3-Gint4.T@BPNSs in the solution was determined by measuring the content of BPNSs within the mixture. After we co-incubated for 6 h, we added CCK-8 reagent, and the absorbance of each well at 450 nm was quantified using a microplate reader (Biotek, USA).

### Stability of Cy3-Gint4.T@BPNSs

Unbound Cy3-Gint4.T was removed by centrifugation at 8,000 rpm for 10 min using a 100-kDa ultrafiltration tube. Pellets were resuspended in PBS and filtered 3 times on days 1, 7, and 14. The remaining filtrate was analyzed by UV–Vis spectrophotometry over 200 to 700 nm. In addition, Cy3-Gint4.T@BPNSs was incubated at 25 and 37 °C, and the fluorescence intensity was measured.

### Knockdown of PDGFRβ by siRNA

Three siRNA sequences (Table S2) were purchased from General Biol (Anhui) Co. Ltd., and Lipofectamine 2000 was obtained from Vazyme Biotech (Nanjing). One day prior to transfection, 7 × 10^5^ U251 cells were seeded in 6-well plates. Following 24 h, siRNA was diluted to 50 nM with 250 μl of Opti-MEM and mixed gently. Lipofectamine 2000 (5 μl) was diluted with 250 μl of Opti-MEM, incubated at room temperature for 6 min, mixed with the siRNA solution, and incubated for another 20 min. The transfection complex (500 μl per well) was added to the 6-well plate, and cells were cultured at 37 °C with 5% CO₂ for 48 h (fresh medium could be replaced 6 h post-transfection).

### Cell passage and cell culture

U87MG, U251, and NHA cells (from the Institute of Cell Biology, Chinese Academy of Sciences) were cultured in DMEM, which was supplemented with 10% fetal bovine serum (FBS; from Gibco, USA) and 1% penicillin–streptomycin, and the cells were grown at 37 °C in 5% CO₂.

### Fluorescence detection

BPNSs were co-incubated with different concentrations of Cy3-Gint4.T (total volume, 100 μl), and fluorescence intensity was quantified at an excitation wavelength of 520 nm using a microplate reader (Bio-Tek, SYNERGY H1, USA). Cells were incubated with phycoerythrin (PE)-labeled Gint4.T antibody for 30 min at 4 °C under light-protected conditions, processed through 2 PBS washes, centrifuged, and reconstituted in 250 μl of PBS prior to fluorescence intensity determination with a flow cytometer (Beckman CytoFLEX, model ZJZXSB-062). Data were analyzed with FlowJo software.

### Fluorescence imaging

U251 cells (5 × 10^5^) were seeded in confocal dishes, allowed to adhere, and incubated with Cy3-Gint4.T@BPNSs or Cy3-R@BPNSs for different durations in the dark at room temperature. Fluorescence was observed using a laser scanning confocal microscope (LSCM; Nikon A1R NIS-Elements 5.4, Japan). For TKI-pretreated cells, 5 × 10^5^ U251 and U87MG cells were treated with TKIs (gefitinib/erlotinib) for 24 h, seeded in confocal dishes, adhered for 4 h, and incubated with Cy3-Gint4.T@BPNSs (C_BPNSs_ = 0.2 mg/ml, C_Cy3-Gint4.T_ = 40 nM) in the dark. Cells were washed 3 times with PBS, fixed with paraformaldehyde for 20 min, and imaged at 561-nm excitation. LSCM data were processed with ImageJ software for quantitative analysis. Quantitative image analysis was performed using ImageJ software. For each confocal field of view, a random single-cell region was selected as the analytical target, and the mean fluorescence intensity of individual cells was calculated.

### Cell apoptosis assay

U251 cells (7 × 10^5^) were seeded in 6-well plates and treated with PBS, siPDGFRβ + TKI, or TKI alone for 48 h. Cells were collected, washed twice with PBS (300*g*, 5 min), resuspended in 500 μl of binding buffer, mixed with 5 μl of Annexin V-FITC and 5 μl of PI, incubated at room temperature in the dark for 5 min, and quantified using a flow cytometer (Beckman CytoFLEX S).

### IC_50_ determination

U251 and PDGFRβ-knockdown U251 (KD U251) cells were seeded in 96-well plates at a density of 5 × 10^4^ cells per well (*n* = 3). After cell adhesion, erlotinib was added at different concentration gradients: 0.1, 0.5, 1, 5, 10, 20, and 50 μM. After co-incubation for 24 h, CCK-8 reagent was added, and the absorbance of each well was measured at 450 nm using a microplate reader (Biotek, USA).

### Statistics

All quantitative experiments were performed in at least 3 independent biological replicates (*n* = 3), each with technical duplicates or triplicates as indicated. All data were analyzed using Origin 2021 software (OriginLab Corporation, USA) and GraphPad Prism software (version 8). For comparisons involving multiple groups, one-way analysis of variance (ANOVA) was applied, whereas 2-group comparisons were performed using the 2-tailed Student’s *t* test. All data are expressed as mean ± SD, and statistical significance was defined as follows: ns (not significant), *P < 0.05, **P < 0.01, ***P < 0.001, and ****P < 0.0001.

## Data Availability

All data generated or analyzed during this study are included in this article and its supplementary files.
