# Supplementary material for: Nano-Enabled Fluorescence Switching: A Novel Strategy for PDGFRβ Detection and TKI Therapy Monitoring
Source: Research (Wash D C). 2026 Mar 24;9:1218. doi: 10.34133/research.1218 (PMC13009536; doi:10.34133/research.1218)
Supplement: Supplementary 1 — Figs. S1 to S10 Tables S1 and S2 [file research.1218.f1.docx]

**Supplementary Information**

**Nano-Enabled Fluorescence Switching: A Novel Strategy for PDGFRβ Detection and TKI Therapy Monitoring**

Xin Fu^1,2†^, Jinyue Fan^2†^, Haoxiang Chen^2†^, Yuli Zheng^2^, Yueqi Liu^1,3^, Chao Zhang^2,4^*, Xiaolong Cao^4,5^*, Tingting Zuo^1^*

^1^College of Biological Sciences and Technology, Yili Normal University, Yining 835000, P.R. China.

^2^Department of Oncology, Zhujiang Hospital, Southern Medical University, Guangzhou 510282, P.R. China.

^3^Xinjiang Key Laboratory of Lavender Conservation and Utilization, Yining 835000, P.R. China.

^4^Translational Medicine Research Center, Zhujiang Hospital, Southern Medical University, Guangzhou 510282, P.R. China.

^5^Department of Anesthesiology, Zhujiang Hospital, Southern Medical University, Guangzhou, Guangdong 510282, P.R. China.

*Address correspondence to: [zc15996256330@smu.edu.cn](mailto:zc15996256330@smu.edu.cn) (C.Z.); [atps@smu.edu.cn](mailto:atps@smu.edu.cn) (X.C.); [ttzuoylsf@163.com](mailto:ttzuoylsf@163.com) (T.Z.).

^†^These authors contributed equally to this work.

**
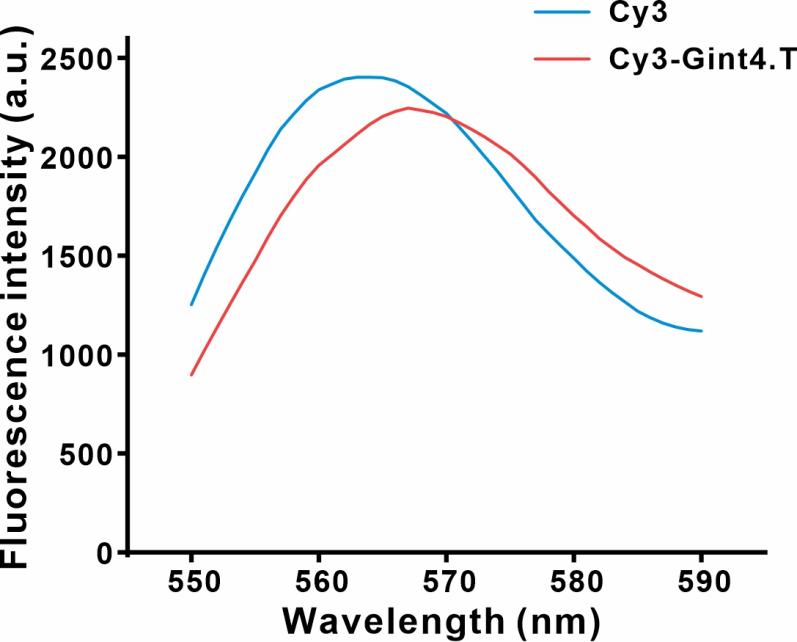
**

**Fig. S1.** Fluorescence spectra of Cy3 and Cy3-Gint4.T.

**
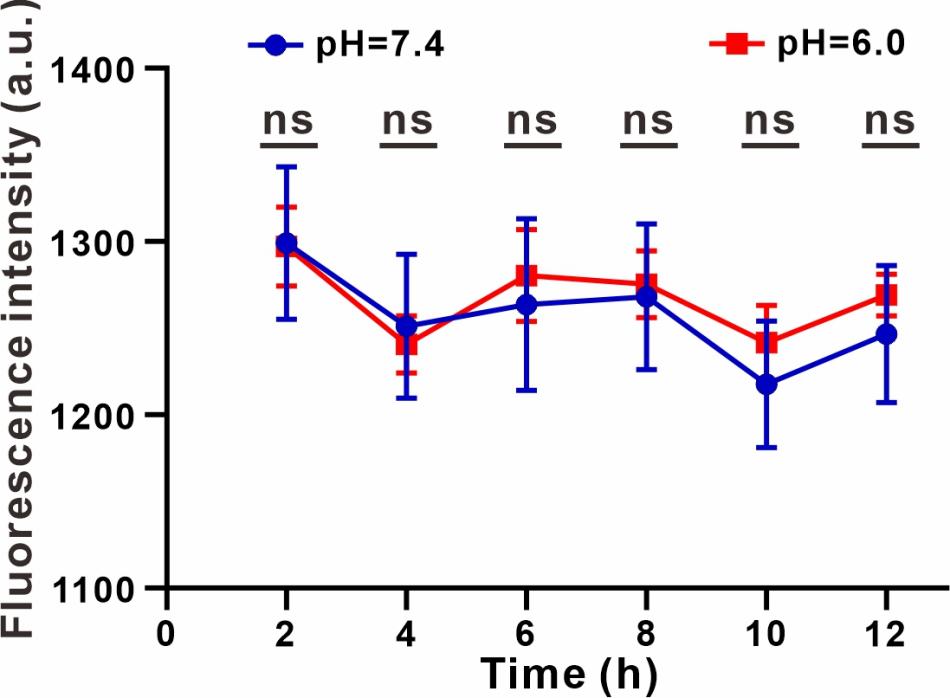
**

**Fig. S2.** Fluorescence stability of Cy3-Gint4.T in pH 6.0 and pH 7.4 PBS buffers at 2, 4, 6, 8, 10 and 12 h. Data are presented as the mean ± SD (n=3, ns means no significance).


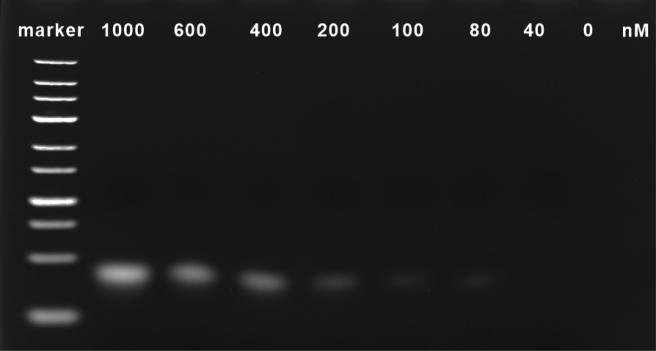


**Fig. S3.** Optimization of the ratio between Gint4.T and BPNSs *via* gel retardation assay: From left to right, the concentrations of Gint4.T are 1000 nM, 600 nM, 400 nM, 200 nM, 100 nM, 80 nM, 40 nM, and 0 nM, with the corresponding concentration of BPNSs kept constant.


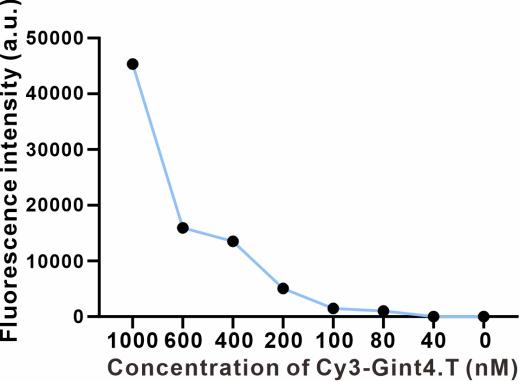


**Fig. S4.** Fluorescence quantitative analysis of gel imaging, corresponding to Fig. S3.


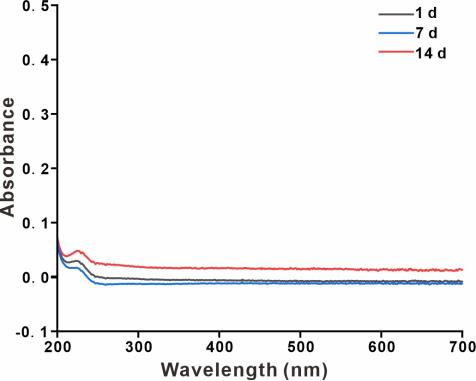


**Fig. S5.** UV absorbance spectra of Cy3-Gint4.T@BPNSs filtrates at day 1, 7 and 14 at room temperature.


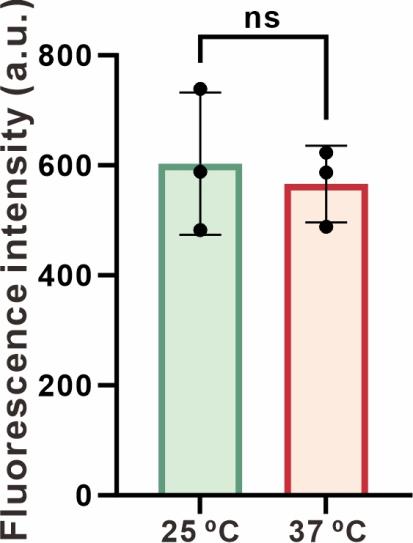


**Fig. S6.** Fluorescence stability of Cy3-Gint4.T@BPNSs at 25 °C and 37 °C. Data are presented as the mean ± SD (n=3, ns means no significance).


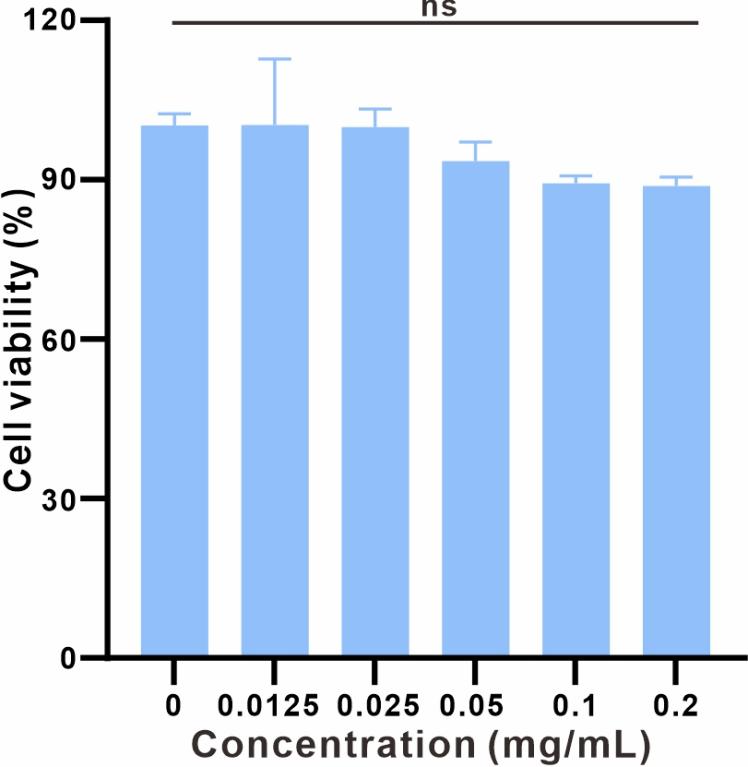


**Fig. S7.** Cell viability of NHA cells after treatment with Cy3-Gint4.T@BPNSs. Data are presented as the mean ± SD (n=3, ns means no significance).


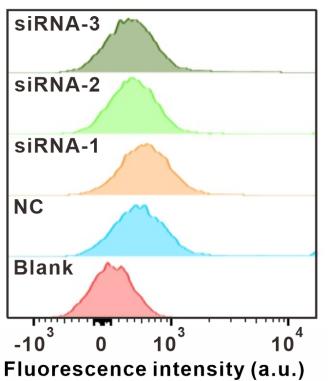


**Fig. S8.** Flow cytometry profiles showing the fluorescence intensity of PDGFRβ in U251 cells after downregulation.


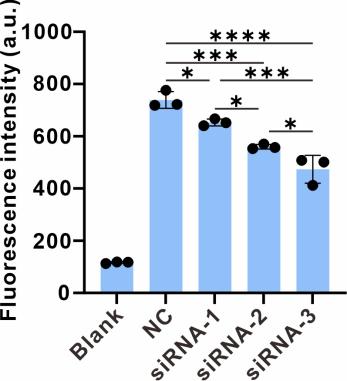


**Fig. S9.** Fluorescence quantitative analysis of flow cytometry profiles, corresponding to Fig. S8. **p* < 0.05, ****p* < 0.001, and *****p* < 0.0001.

**
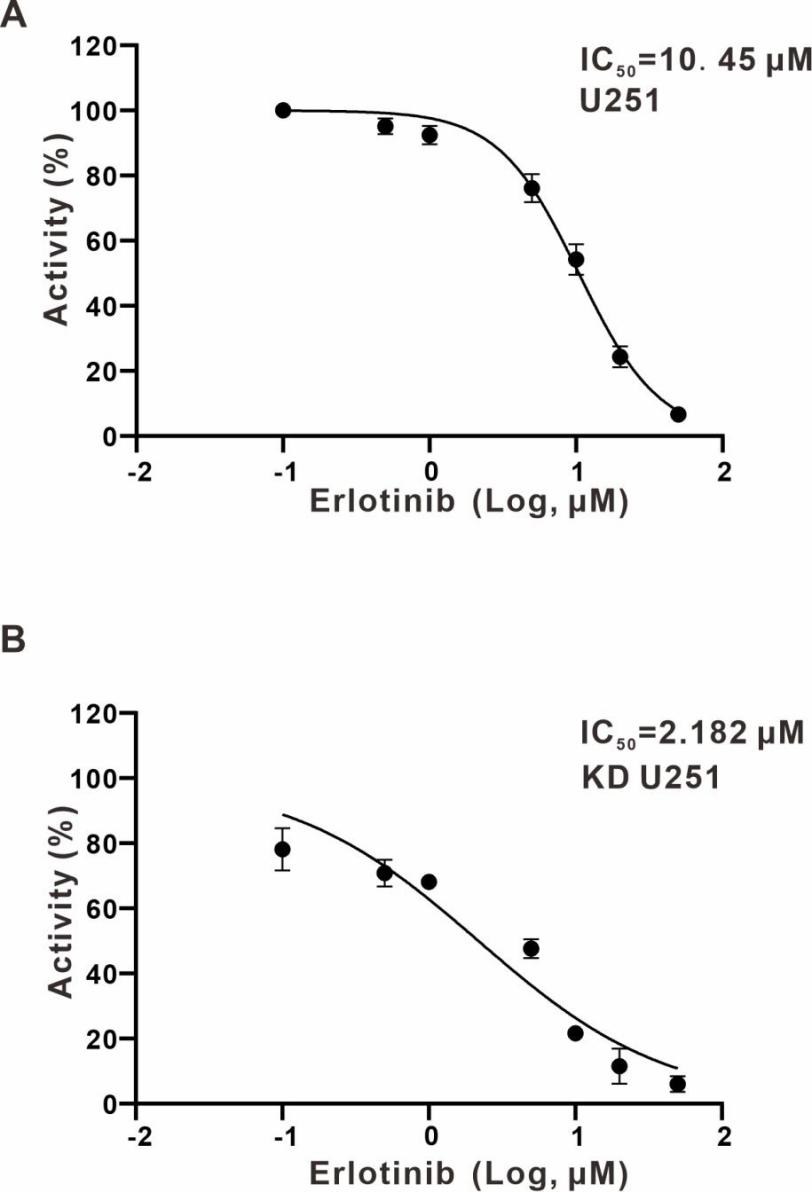
**

**Fig. S10.** The erlotinib IC_50_ curve graph of (A) U251 (B) KD U251.

**Table S1.** Cy3-Gint4.T and Cy3-R sequences.

| Type | Sequences (5’-3’) |
| --- | --- |
| Cy3-Gint4.T | Cy3-UGUCGUGGGGCAUCGAGUAAAUGCAAUUCGACA |
| Cy3-R | Cy3-UUCGUACCGGGUAGGUUGGCUUGCACAUAGAACGUGUCA |

**Table S2.** The siRNA sequences (5’ to 3’).

| siRNA-1 | /rG//rG//rA//rA//rU//rG//rA//rG//rG//rU//rG//rG//rU/rC//rA//rA//rC//rU//rU/TT |
| --- | --- |
| siRNA-2 | /rU//rU//rG//rC//rU//rG//rU//rA//rG//rA//rG//rC//rU//rC//rC//rG//rC//rG//rC/TT |
| siRNA-3 | /rA//rC//rG//rG//rA//rG//rA//rC//rC//rU//rG//rG//rU//rG//rG//rA//rC//rU//rA/TT |
